# Sloped Terrain Segmentation for Autonomous Drive Using Sparse 3D Point Cloud

**DOI:** 10.1155/2014/582753

**Published:** 2014-06-24

**Authors:** Seoungjae Cho, Jonghyun Kim, Warda Ikram, Kyungeun Cho, Young-Sik Jeong, Kyhyun Um, Sungdae Sim

**Affiliations:** ^1^Department of Multimedia Engineering, Dongguk University-Seoul, Seoul 100-715, Republic of Korea; ^2^Agency for Defense Development, Daejeon 305-152, Republic of Korea

## Abstract

A ubiquitous environment for road travel that uses wireless networks requires the minimization of data exchange between vehicles. An algorithm that can segment the ground in real time is necessary to obtain location data between vehicles simultaneously executing autonomous drive. This paper proposes a framework for segmenting the ground in real time using a sparse three-dimensional (3D) point cloud acquired from undulating terrain. A sparse 3D point cloud can be acquired by scanning the geography using light detection and ranging (LiDAR) sensors. For efficient ground segmentation, 3D point clouds are quantized in units of volume pixels (voxels) and overlapping data is eliminated. We reduce nonoverlapping voxels to two dimensions by implementing a lowermost heightmap. The ground area is determined on the basis of the number of voxels in each voxel group. We execute ground segmentation in real time by proposing an approach to minimize the comparison between neighboring voxels. Furthermore, we experimentally verify that ground segmentation can be executed at about 19.31 ms per frame.

## 1. Introduction

The objective of this study is to develop a system that accurately plans travel routes for an unmanned autonomous vehicle on the basis of the vehicle's driving data and geographical location. To this end, datasets are acquired through a variety of sensors, and data required for route planning is extracted from these datasets. The route is designed to avoid obstacles, by accurately identifying different kinds of obstacles on the road, in order to assist an unmanned vehicle to successfully arrive at its destination. At present, ground segmentation technology is a necessary preprocessing step to identify roads available for driving as well as obstacles on the roads, based on the vehicle's geographical information.

Vehicle-to-everything (V2X) technologies have been actively investigated in order to develop intelligent vehicles. These technologies include the autonomous driving technology explained above. V2X is designed to improve traffic safety and provide a variety of services to drivers by combining wireless communication technologies, such as vehicle-to-vehicle (V2V), vehicle-to-infrastructure (V2I), and vehicle-to-nomadic (V2N) technologies [1]. A systematic travel route can be planned using these V2X technologies by sharing the vehicle's geographical information. Data traffic is reduced to facilitate communication using wireless network [2, 3].

Autonomous drive in various geographical environments requires the technology to accurately segment the ground even in slopes. Vehicles, such as those involved in military operations in mountainous areas, frequently have to negotiate a series of steep hills. Since the position of a vehicle rapidly changes in such an environment, it is not easy to accurately segment the ground. For reliable travel route planning on slopes, our proposed system will conduct ground segmentation in real time.

Sensors are required to detect the surrounding geography for accurate ground segmentation. Thus, a light detection and ranging (LiDAR) sensor is used to accurately detect geographical shapes. LiDAR sensors calculate the distance between the vehicle and the surrounding surface by emitting a number of lasers. The launch angle of the laser and the distance data are converted into three-dimensional (3D) points. The number of points acquired ranges from thousands to hundreds of thousands. For LiDAR, on account of mass production at low cost, the density of the 3D point cloud acquired is low. Thus, it is difficult to accurately segment the ground. Accordingly, when LiDAR is used in our system, 3D point clouds will be continuously accumulated and overlapping points will be eliminated. The 3D point clouds are accumulated through a simultaneous localization and mapping (SLAM) algorithm, or by using a differential global positioning system with inertial measurement unit (D-GPS/IMU) sensor. To accurately eliminate overlapping data, the 3D point clouds need to be operated in a one-coordinate system.

In order to satisfy the above requirements, we propose a ground segmentation framework based on a wireless network environment to successfully plan travel routes in real time in a variety of geographical environments. Our framework builds a dynamic flagmap data structure to reduce the amount of data by eliminating overlapping data and is comprised of several algorithms that execute ground segmentation based on certain data structures. This study can be applied to the navigation of autonomous agriculture vehicles and indoor robots [4, 5].

In Section 2, we summarize related research in the area. We present our ground segmentation framework in Section 3.  Section 4 describes dynamic flagmap implementation for effective ground segmentation, whereas Section 5 explains the ground segmentation algorithm executed on the basis of the dynamic flagmap. In Section 6, we describe an experiment to verify the efficiency of our framework. We offer concluding thoughts in Section 7.

## 2. Related Work

Ground segmentation technologies have been investigated in a variety of fields, including autonomous drive, 3D geography modeling, and object tracking.

Moosmann et al. proposed an approach to segment the ground as well as objects on the basis of a local convexity criterion [6]. This approach cannot be applied for LiDAR which detects low-density 3D point clouds because it uses LiDAR detecting high-density 3D point clouds. Furthermore, Moosmann et al.'s system cannot process data at the same time of acquiring scanned data because of high operation cost by the algorithm.

Research in the area was conducted that did not apply general approaches based on relationships with neighboring data and features of points [7]. The research proposed quick segmentation technology by applying a two-dimensional (2D) line extraction algorithm to 3D points. The approach can be applied to mild slopes, but its efficiency in environments with tortuous and continually undulating routes has not been verified. Moreover, since 3D point clouds acquired through LiDAR have high density, the approach cannot be applied to low-density 3D point clouds.

Douillard et al. proposed different object segmentation approaches for high-density and low-density 3D point clouds [8]. They implemented a framework that executes ground segmentation and then clusters the data. For low-density 3D point cloud data, they used Gaussian process incremental sample consensus algorithm to estimate the ground. However, this approach is not appropriate for autonomous drive because it requires that users select points that are certainly on the ground as seeds.

Other research papers focused on converting 3D point clouds from LiDAR to 2D range images and on segmenting objects from them [9]. The approach cannot be applied to slopes because it uses the simple threshold method assumption that the ground is flat when eliminating ground data, during the preprocessing step for object segmentation.

Song et al. carried out ground segmentation using height histograms and a Gibbs-Markov random field model to reconstruct the geography into a 3D model [10]. However, the efficiency of its algorithm for slopes has not been verified.

Chen et al. have proposed a real-time ground segmentation approach for travel route planning of autonomous land vehicle in open space [11]. The approach applies one-dimensional (1D) Gaussian process regression to the basis of a circular polar grid map. The approach cannot execute ground segmentation on steep slopes.

The problems identified by investigating existing approaches to ground segmentation are summarized as follows.

- Ground segmentation cannot be executed in low-density 3D point clouds.
- Ground segmentation cannot be executed for geographical features that have uphill and downhill roads.
- Ground segmentation cannot be executed in real time.

Accordingly, we propose in this paper a ground segmentation framework that solves the above problems. The framework comprises ground segmentation technologies to plan a travel route in real time, using a wireless network environment, in a geographical environment containing a several hills.

## 3. Overview of Ground Segmentation Framework

LiDAR and D-GPS/IMU are installed on an autonomous vehicle to obtain 3D surface information and vehicle movement information, respectively. For the sparse 3D point cloud acquired from LiDAR, it is difficult, from one point, to acquire information about neighboring points because of the long distance between points.

**Figure 1:**
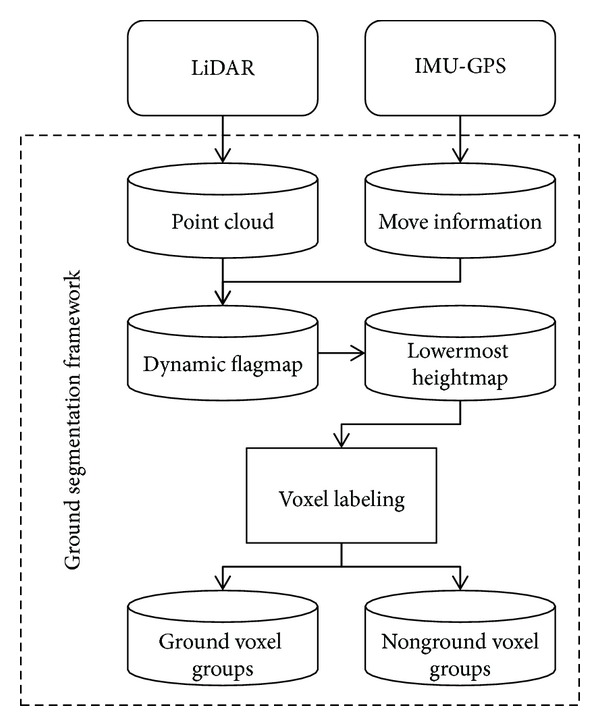
Overview of the ground segmentation framework.

For accurate ground segmentation, the sparse 3D point cloud needs to be accumulated continuously during the vehicle's movement. Accordingly, data size becomes larger with time. The size of the data needs to be reduced in order for it to be transferred through the wireless network. Consequently, the efficiency of the algorithm improves as the data size is reduced. We propose a ground segmentation framework comprising various algorithms, including ones for the elimination of overlapping data and ground segmentation.  Figure 1 illustrates the ground segmentation system proposed in this paper.

The framework performs ground segmentation through the following three steps:

- dynamic flagmap implementation to eliminate overlapped data,
- lowermost heightmap implementation to improve efficiency of ground segmentation algorithm,
- voxel labeling algorithm implementation for grouping neighboring voxels.

To reduce the number of points in 3D point clouds with a lot of data, overlapping points should be deleted as the vehicle moves. In the interest of this, this paper proposes a dynamic flagmap data structure expressing the volume pixel (voxel) space quantized in 10 cm units. The implementation of the dynamic flagmap data structure helps eliminate points assigned to the same voxel.

The next step is effectively identifying the driving route of the autonomous vehicle. Ground segmentation is performed based on the dynamic flagmap, which comprises nonoverlapping voxels. Several other algorithms are also applied in concert.

The implementation of the lowermost heightmap helps reduce the data size required for the ground segmentation algorithm and removes a number of nonground voxels. Heightmap is a general approach to effectively represent a terrain and is based on a two-dimensional coordinate system comprising only the *x*-axis and the *z*-axis. Each 2D coordinate has a constant height value. The lowermost heightmap is used for preprocessing in order to select only voxels with high ground-segmentation probability. The reduction in the number of voxels used in subsequent algorithms improves their efficiency.

Once the lowermost heightmap has been built, the neighboring voxels are gathered by a voxel labeling algorithm. The algorithm assigns the same label to voxels with a small difference in height with respect to the lowermost heightmap. When labeling for all voxels is complete, the position of each voxel is determined, that is, whether or not each voxel is on the ground. The algorithm to reduce access to neighboring voxels is applied to reduce the execution time of the algorithm.

## 4. Building Dynamic Flagmap

The 3D point cloud acquired from LiDAR is represented using local coordinates based on the sensor. As the autonomous vehicle with LiDAR mounted on it moves, the overlapping points among the local 3D point clouds acquired per frame cannot be properly identified. Thus, we need to convert and accumulate local 3D point clouds acquired per frame into a global coordinate system. For this, the vehicle's movement information is acquired by the D-GPS/IMU mounted on it. Once the 3D point clouds are integrated into a single coordinate system, the neighboring points can be removed as they are determined to be overlapping points.

However, the estimation of adjacency between 3D points that have real number values requires more calculation than 2D points. In this section, we propose a data structure, called dynamic flagmap, that finds overlapping points by easily identifying adjacency between two points. To present the voxel space “flagmap” simply, the voxel location is expressed using a 1D array, and not 3D coordinates. Each element in the array is one-bit Boolean data item to indicate the voxel's existence. Such approach facilitates access between neighboring points.

To reduce the dimensions of the 3D voxels and to express them in a 1D array, we need to reduce voxel space. This is because the array size, which can be expressed by the array index, is limited, and large array data requires more memory. The following equation is used to convert a local 3D point into an array index item in a limited voxel space [12]:

(1) v=2WH·floor(zμ+D2)+H·floor(xμ+W2)+floor(yμ+H2),

where *W*, *H*, and *D* represent the width, height, and depth in a limited voxel space, respectively, *x*, *y*, and *z* are the values of the *x*-axis, the *y*-axis, and the *z*-axis of each local 3D point, respectively, and *μ* represents the length of a side of each voxel and *v* is an array index. The equation above helps easily reduce memory usage and estimate adjacency among voxels by quantizing a local 3D point and converting it into an array index.

**Figure 2:**
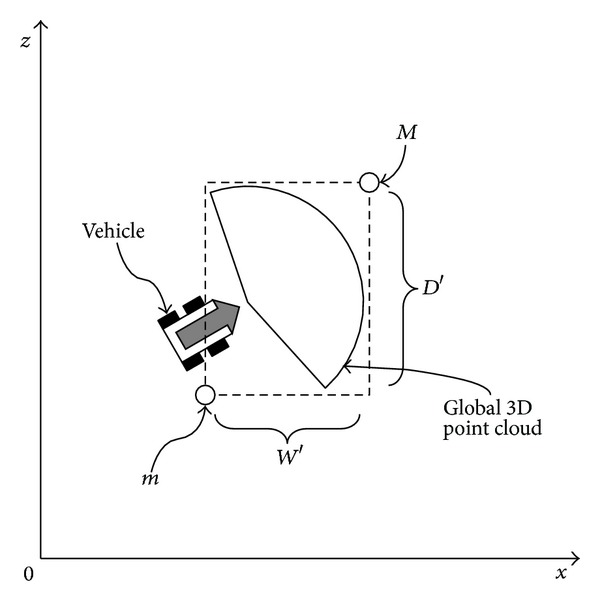
Structure to express a local 3D point cloud in a global coordinate system in a fixed memory (from top view).

However, since (1) determines whether or not a point is overlapping based on a local 3D point, it cannot be used if an autonomous vehicle moves at high speed. To determine data overlap in such a case, we implement the dynamic flagmap on the basis of 3D global points.  Figure 2 shows the structure required to express local 3D point clouds acquired per frame on a global coordinate system in the fixed memory.

In Figure 2, *W*′, *H*′, and *D*′ represent the width, height, and depth of the dynamic voxel space, respectively. They are indicated in 2D from the top view, so that *H*′ is omitted. *m* and *M* are the bounds, respectively, of dynamic voxel space calculated with the minimum and the maximum value of the *x*′, *y*′, and *z*′-axes among the 3D global point clouds acquired per frame. Both values are dynamically changed depending on the direction of the autonomous vehicle. Furthermore, *W*′, *H*′, and *D*′ vary with* m* and* M*. The maximum values of *W*′, *H*′, and *D*′ are determined depending on the maximum distance in the 3D point cloud acquired from LiDAR. Accordingly, the array size of the dynamic flagmap is determined according to the maximum value of *W*′, *H*′, and *D*′ as shown in the equation below:

(2) $$\begin{array}{c} \text{size}=\max W^{\prime }\times \max H^{\prime }\times \max D^{\prime }. \end{array}$$

Voxel space around the vehicle given in the array with a fixed size can be expressed in global coordinates by updating *m* and *M* per frame. In other words, the coordinates of the 0th index in the array of the dynamic flagmap are the same as* m*. The coordinates of the (*W*′ × *H*′ × *D*′ − 1)th index are identical to* M*. The following equation converts a 3D point into an array index using the dynamic flagmap:

(3) v′=2W′H′·floor((zm−z′)μ+D′2)+H′·floor((xm−x′)μ+  W′2)  +floor((ym−y′)μ+  H′2).

The global coordinates can be expressed in the array with the fixed size by expressing the location of voxel based on the dynamic flagmap as described above.

The following supplementary process is required to determine whether voxels converted into global coordinates overlap. Let us express* m* and* M* at time *t*_*i*_ as *m*_*i*_ and *M*_*i*_, respectively. Then, *m*_*i*_ ≠ *m*_*j*_ and *M*_*i*_ ≠ *M*_*j*_, if *i* ≠ *j*. This is because of an error in vehicle location even when an autonomous vehicle stops. Accordingly, all 3D global points are kept in a separate linked list after they have been converted into an array index and added to the dynamic flagmap. Each 3D global point in the linked list is deleted when it exceeds the bounds of dynamic voxel space per frame. As the 3D global points are kept in the linked list, voxel overlap can be determined based on the global coordinate system.

In this section, we explained the dynamic flagmap data structure that can effectively eliminate overlapping data. The data structure helps reduce data size required for ground segmentation and thus improves the performance of the algorithm.

## 5. Ground Segmentation

Ground segmentation is a preprocessing step in planning the route of an autonomous vehicle. Ground segmentation requires a dynamic flagmap, which consists of a 1D Boolean array and bounds of dynamic voxel space. Using the dynamic flagmap, a lowermost heightmap is constructed. The voxel labeling algorithm is executed using the lowermost heightmap. Ground voxel groups and nonground voxel groups are generated because of ground segmentation. This section describes an effective and efficient ground segmentation approach for an autonomous vehicle.

### 5.1. Lowermost Heightmap

A lowermost heightmap is the basic data structure for the execution of ground segmentation. We show how to improve the estimation efficiency of the ground segmentation mechanism and reduce the number of nonground voxels by building a lowermost heightmap. A heightmap generally consists of a plane coordinate system with the *x*-axis and the *z*-axis, such that each coordinate has a height value. We reduce the dimensions of 3D voxels to 2D by using the above structure. The 3D point cloud acquired from LiDAR is the distance data between a nearby object surface and the autonomous vehicle. The 3D point cloud cannot detect the object surface, which is lower than the ground. A lowermost heightmap considers account for such a scenario and is thus comprised of only voxels with the smallest height values.  Pseudocode 1 illustrates the algorithm that builds the lowermost heightmap using a dynamic flagmap.

**Pseudocode 1:**
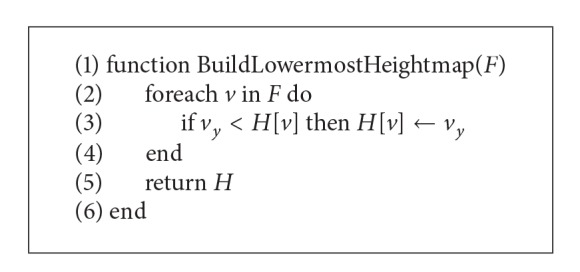
Building lowermost heightmap.

In the algorithm, *F* is the dynamic flagmap, *v* is the index in the array, *v*_*y*_ is the height value of *v* on the *y*-axis, and *H* is the lowermost heightmap. The lowermost heightmap has only one height value on the vertical line passing each (*x*, *z*) coordinate on the *xz* plane. Accordingly, the height value of neighboring (*x*, *z*) coordinates of a specific (*x*, *z*) coordinate can be immediately identified.

### 5.2. Voxel Labeling

The ground surface on which an autonomous vehicle moves has features similar to a sloped continuous surface. This feature of the ground surface is roughly modeled by building a lowermost heightmap. However, a lowermost heightmap also includes a height value, which does not exist on the ground surface. Voxel labeling is an algorithm that creates voxel groups by classing together geometrically continuous voxels among neighboring voxels. In this section, we propose an approach to minimize access to neighboring voxels for voxel labeling in real time.

Voxel labeling is based on the lowermost heightmap. The lowermost heightmap can access voxels neighboring each voxel very quickly because each voxel position is an array index.

When the height difference between two neighboring voxels is below a certain threshold, the same label is assigned to both voxels, and hence one voxel group is created. When the height difference between neighboring voxels is in the ±1 range, both voxels have a high probability of being part of the ground. Thus, they are placed in the same group. The bounds of each voxel group expand as voxel labeling proceeds. If even one voxel in a group is close in value to any voxel in another group, the two groups are integrated. Voxel labeling applies an optimized algorithm for effective performance in real time.  Pseudocode 2 illustrates the voxel labeling algorithm.

**Pseudocode 2:**
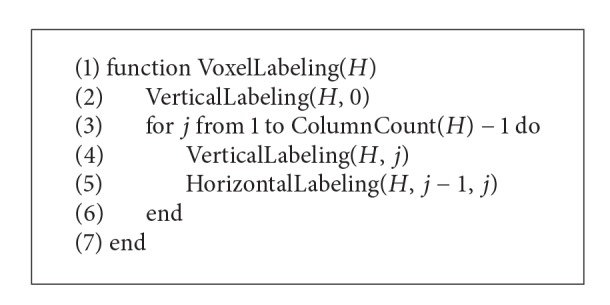
Voxel labeling algorithm.

The algorithm considers the lowermost heightmap as a kind of matrix equation. The following equation defines the row and column in a lowermost heightmap:

(4) $$\begin{array}{c} {\text{row}}_{i}=\left\{\forall x,z=i\;|\;\left(x,z\right)\right\},\\ {\text{column}}_{j}=\left\{\forall x,z=j\;|\;\left(x,z\right)\right\}. \end{array}$$

**Figure 3:**
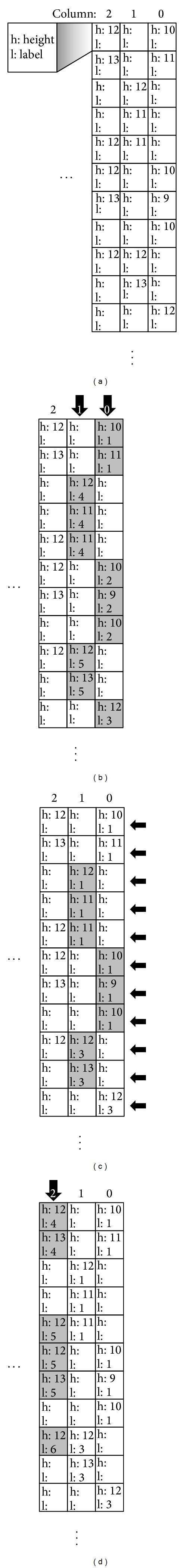
Voxel labeling in lowermost heightmap. Each square represents a voxel, which has height and label attributes. (a) Initial state of lowermost heightmap before executing voxel labeling. (b) Execute VerticalLabeling for 0th and 1st columns independently. (c) Execute HorizonalLabeling for the two columns. (d) After executing VerticalLabeling for 2nd column, execute (c) for 1st and 2nd columns.

**Figure 4:**
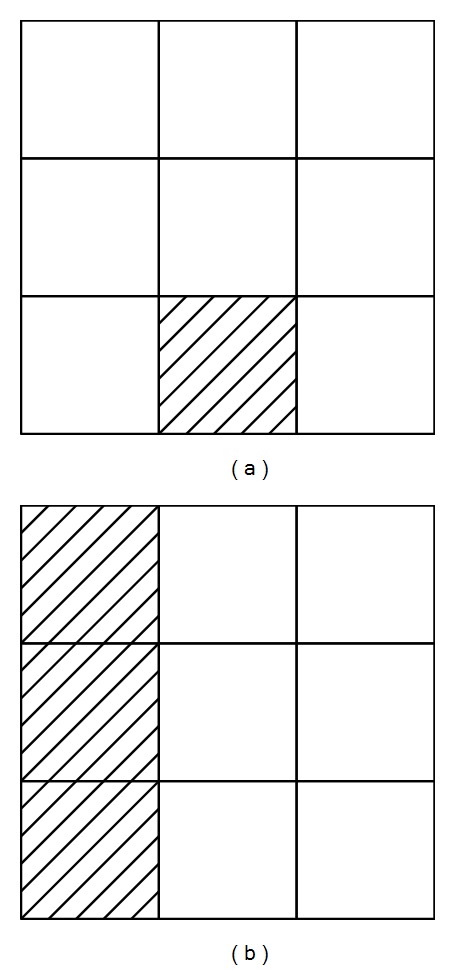
Neighboring voxels, to which each voxel needs access for voxel labeling. (a) Neighboring voxels in vertical labeling. (b) Neighboring voxels in horizontal labeling.

Figure 3 describes the voxel-labeling process. The figure illustrates the conceptual top view of the lowermost heightmaps. First, vertical labeling is applied to voxels in the *j*th and *j* + 1th column in the lowermost heightmap. Vertical labeling is the process to label one column. When the height difference between voxels at (*i*, *j*) and (*i* + 1, *j*) is in the ±*τ* range, both voxels are labeled as part of the same group. The process is repeated for all rows in the *j*th column. Vertical labeling is also executed on the *j* + 1th column. Finally, voxel groups are generated for each column.  Figure 4(a) illustrates the neighboring voxels that each voxel needs to access in order to compare height difference in vertical labeling. Following this, voxel groups in both columns are integrated by horizontal labeling. When the height difference between each voxel in the *j*th column and in the neighboring *j* + 1th column is below ±*τ*, horizontal labeling integrates both voxel groups.  Figure 4(b) shows the neighboring voxels.

When both columns have been integrated, vertical labeling is executed on the voxels in the *j* + 2th column and horizontal labeling on the *j* + 1th and *j* + 2th columns. The process is repeated until voxel labeling has been executed for all columns in the lowermost heightmap. The voxel group with the highest number of voxels is determined to be the final ground voxel group.

## 6. Experiments

We performed an experiment to verify the efficiency of our ground segmentation framework. For the experiment, 3D point clouds were acquired from actual mountainous roads approximately 3.5 km in length, which consisted of flatlands, slopes, trees, and buildings. The experiment platform is a vehicle on which LiDAR was mounted. Using the sensor, the vehicle acquired dataset at an average velocity of about 20 km/h. The LiDAR model used for the experiment is Velodyne HDL-32E. To increase the density of the 3D point clouds, we only used data within about 30 m of the LiDAR. The PC used for ground segmentation had an Intel i7-870 (CPU) and a DDR3 10600 8 GB (RAM). The algorithm was tested on the acquired dataset.

**Figure 5:**
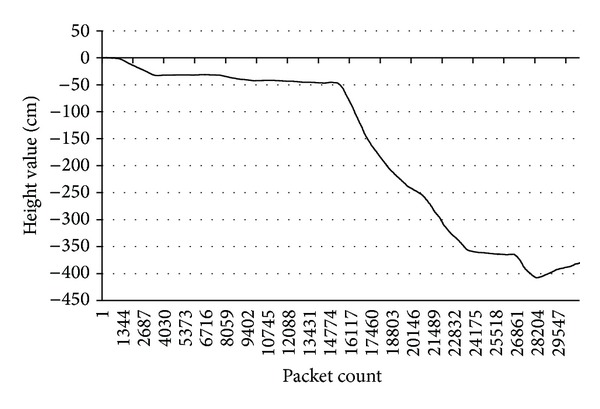
Slope of the geography used in the experiment.

Local 3D point clouds from LiDAR were accumulated based on global coordinates using the vehicle's movement information.  Figure 5 illustrates the slope of the geographical area used in the experiment.

Figure 8 visualizes the ground segmentation results. The results were visualized with textured mesh and colored points to represent ground and nonground, respectively. Color of each point was assigned according to the height of the point.

To validate the performance of proposed framework, we compared it with threshold based segmentation method. Threshold based method uses a height value to classify voxels into ground and nonground voxels. Figures 8(a), 8(c), 8(e), 8(g), and 8(i) are the results by threshold based method. And the results by ground segmentation framework proposed in this paper are illustrated in Figures 8(b), 8(d), 8(f), 8(h), and 8(j). There is no big difference between Figures 8(a) and 8(b), because the parts of the scene were captured at a flat road. In Figure 8(c), a slope at right side was misclassified as nonground. But, in Figure 8(d), the slope was classified as ground correctly.  Figure 8(e) illustrates that some parts of trees are rendered as textured mesh, because they were misclassified as ground. Whereas in Figure 8(f), the parts of trees were rendered as colored points by classifying them as nonground. Voxels in an uphill road were misclassified as nonground in Figures 8(g) and 8(i). But they were classified properly as ground in Figures 8(h) and 8(j).

**Figure 6:**
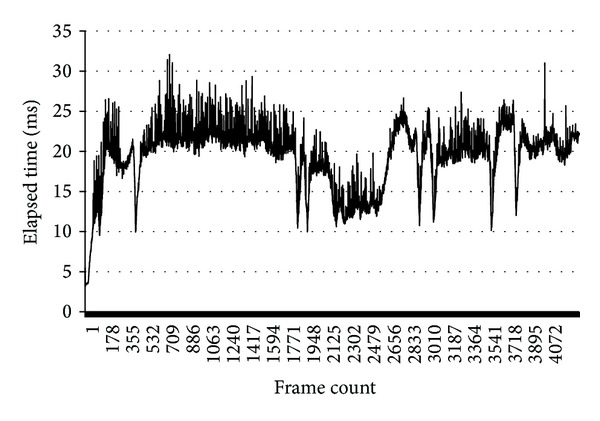
Elapsed time performing segmentation.

The LiDAR used for the experiment typically scans the surrounding environment at a rate of about 10 Hz. To verify ground segmentation in real time, we executed ground segmentation at about 20 Hz, twice as quick as the rotation speed of the LiDAR.  Figure 6 indicates the time spent on ground segmentation per ground segmentation frame in the graph.  Table 1 shows the time required for ground segmentation per frame. It verifies that the proposed framework is enough to segment ground at real time, as the average elapsed time performing segmentation is less than 50 ms. 50 ms is the maximum limitation to perform segmentation at 20 Hz.

**Figure 7:**
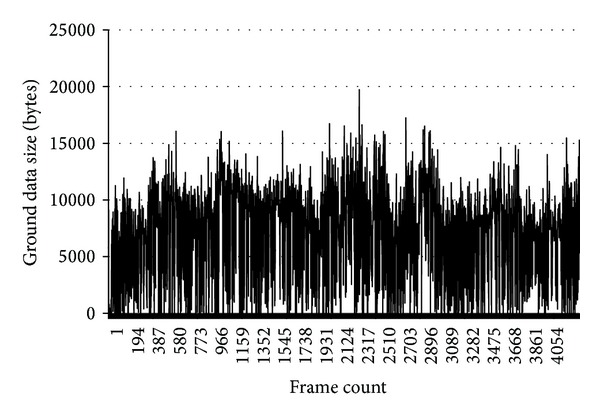
Ground data size after segmentation.

**Figure 8:**
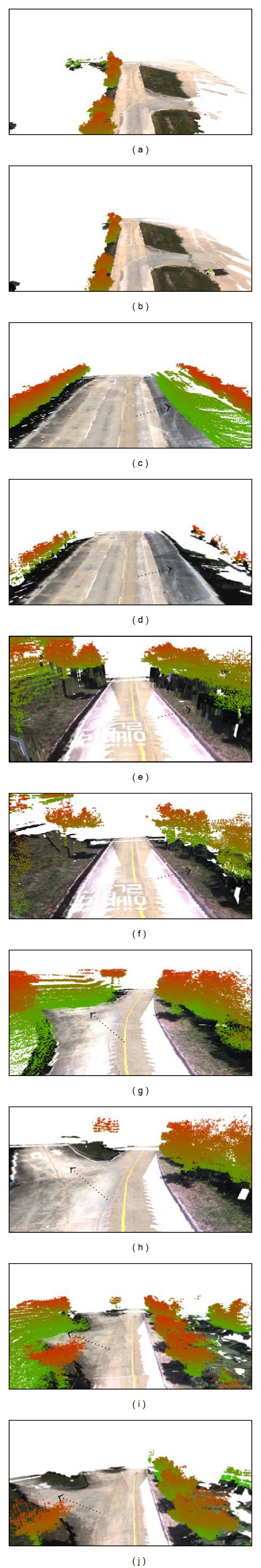
Ground segmentation results (the different parts are indicated by arrows). (a) Threshold based method result at a flat road. (b) Proposed method result at a flat road. (c) Threshold based method result at flat road with a slope. (d) Proposed method result at flat road with a slope. (e) Threshold based method result at a downhill road. (f) Proposed method result at a downhill road. (g) Threshold based method result at an uphill road. (h) Proposed method result at an uphill road. (i) Threshold based method result at another uphill road. (j) Proposed method result at another uphill road.

Figure 7 represents the ground data size following ground segmentation by frame in the graph.  Table 2 shows the ground data statistics. Because the segmentation was performed at 20 Hz in this experiment, average ground data size (bytes) by frame can be converted to Mbps unit using this equation:

(5) $$\begin{array}{c} \text{Mbps}=\frac{\left(\text{bytes}\times 8\times 20\right)}{1024^{2}}. \end{array}$$

With this equation, average Mbps is calculated as 1.14 Mbps. Because minimum data rate per stream of 802.11a network standard is 6 Mbps, the data size is acceptable to typical wireless network.

## 7. Conclusion

In this paper, we proposed a ground segmentation framework for real-time route planning through a wireless network for an autonomous vehicle in a ubiquitous road environment. The framework involves elimination of overlapping data, the reduction of data dimensions, and ground segmentation. To this end, the implementation of the dynamic flagmap, the lowermost heightmap, and technologies including voxel labeling were described in detail. A voxel labeling algorithm was developed to minimize access to neighboring voxels for real-time ground segmentation. Furthermore, we experimentally verified the efficiency of our real-time ground segmentation system, even in a geographical environment with numerous hills. The segmented ground data size can be shared through a wireless network in real time by binary compression.

Our future research will deal with technology for eliminating small objects along a road, such as bushes, from the ground data. To this end, we will develop an algorithm that considers the density of the voxel group in real time.

## Figures and Tables

**Table 1:** Stats of elapsed time by frame.

Average elapsed time	19.31 ms
Maximum elapsed time	32.07 ms
Standard deviation	3.91 ms

**Table 2:** Stats of ground data size by frame.

Average data size	7467.57 bytes
Maximum data size	19716.00 bytes
Standard deviation	3591.44 bytes
